# Together we unite: the role of the Commonwealth in achieving universal health coverage through pharmaceutical care amidst the COVID-19 pandemic

**DOI:** 10.1186/s40545-020-00214-6

**Published:** 2020-05-13

**Authors:** Amy Hai Yan Chan, Victoria Rutter, Diane Ashiru-Oredope, Chloe Tuck, Zaheer-Ud-Din Babar

**Affiliations:** 1 Commonwealth Pharmacists Association, London, UK; 2 School of Pharmacy, Faculty of Medical and Health Sciences, University of Auckland, Level 3, Building 505, 85 Pard Road, Grafton, Auckland, 1023 New Zealand; 3 School of Health and Related Research (ScHARR), University of Sheffield, Sheffield, UK; 4 University of Huddersfield, Huddersfield, UK

**Keywords:** Pharmacy, Pharmacists, Commonwealth, Health, Heads of government, Pandemic, CHOGM

## Abstract

The world currently faces unprecedented health challenges as COVID-19 poses a huge threat to health systems, economies and societies as we know it. The events of the current COVID-19 pandemic have further emphasised existing issues within our health systems. There is no better time than now to come together in global solidarity to tackle these evolving threats of COVID-19 pandemic. The Commonwealth is an ideally placed network to tackle these global health challenges, with its wide-reaching networks of governmental, non-governmental and civil society organisations across all continents. Although the biennial Commonwealth Heads of Government Meeting (CHOGM) originally scheduled to take place in Kigali in Rwanda 22–27 June 2020 has been postponed in view of COVID-19, Commonwealth country discussions are continuing, centred on the CHOGM key theme of ‘Delivering a Common Future: Connecting, Innovating, Transforming’, and five subthemes of Information and Communications Technology (ICT) and Innovation; Trade; Environment; Governance and the Rule of Law; and Youth. The planned CHOGM and Commonwealth itself provides all members a timely platform to consider innovative ways to connect, innovate and transform healthcare to meet the needs of their populations. This commentary considers these five CHOGM subthemes and how member nations can be supported to achieve universal health coverage through optimising medicines use and outcomes, in the midst of a global pandemic in line with the global health agenda.

The year 2020 marks the beginning of a new decade, and with it a new set of challenges but also opportunities. The biennial Commonwealth Heads of Government Meeting (CHOGM) was scheduled to take place in Kigali in Rwanda 22–27 June 2020, however, has been postponed indefinitely in view of COVID-19 [1]. Despite the postponement, virtual forums and discussions centred on the key theme for CHOGM 2020 – ‘Delivering a Common Future: Connecting, Innovating, Transforming’ [2] – are ongoing. These discussions are particularly pertinent in the COVID-19 pandemic to ensure that the rapidly changing needs of member countries are met. When the theme for this meeting was conceived, no one imagined how relevant and timely it would be in these current times of the COVID-19 global health crisis. The events of the pandemic have only served to further emphasise existing issues within our health systems that were highlighted at the last CHOGM [3] such as substandard and falsified medication [4], and concerns about the health workforce shortages [5, 6]. Additionally, the COVID-19 pandemic has brought new, unprecedented health and ethical challenges [7] such as questions on how to ensure ongoing, equitable access to pharmaceutical care [8], and protect the safety of our current health workforce [9]. Yet, with this comes a great need and motivation to evolve innovative approaches to communicate and collaborate in response to these new pressures. There is no better time than now to bond together in global solidarity to tackle these emerging threats of COVID-19 pandemic [10].

The Commonwealth is a key multilateral organisation, comprising 54 member nations spread across Africa, Asia, the Americas, Europe and the Pacific, encompassing many lower-middle income countries (LMICs) [11]. It includes associated networks consisting of a myriad of governmental, non-governmental and civil society organisations working together to deliver collaborative actions based on shared objectives. The Commonwealth is thus a ready-made network spanning a diverse range of countries, making it ideally placed to help tackle the global challenge of COVID-19 [11]. The upcoming CHOGM meeting is a timely platform to address these issues.

Five sub-themes have been identified for discussion at CHOGM 2020: Information and Communications Technology (ICT) and Innovation; Trade; Environment; Governance and the Rule of Law; and Youth [2]. These discussions will build on the progress that was achieved since the last CHOGM 2018 in London. The discussions are expected to centre on the ways that a contemporary Commonwealth can transform societies, in line with the Commonwealth Charter values of democracy, multilateralism, sustainable development, and empowerment of women and youth, whilst considering the impact of the COVID-19 pandemic.

In these rapidly changing times, countries around the world, both within and outside of the Commonwealth, have been faced with unprecedented health system pressures and human rights challenges that have prompted the development of innovative ways to connect, innovate and transform healthcare to meet the needs of their populations [7]. High on the list of priorities to address is the issue of pharmaceutical access and quality pharmaceutical care [8]. Whilst all of the United Nations Sustainable Development Goals (SDGs) are affected by COVID-19 [10], SDG 3: Good health and wellbeing is the most directly impacted. As medicines are the most common and significant intervention in our society [12], ensuring equitable ongoing medicines access is key for achieving universal health coverage (UHC).

The Commonwealth Pharmacists Association (CPA) is an accredited organization of the Commonwealth and an active member of the Commonwealth Health Profession’s Alliance (CHPA), who advocates for all aspects of health to national policy makers and Commonwealth governments. Representing the pharmacy workforce, the CPA’s mission is to ensure safe and effective medicines use to improve health and well-being throughout the Commonwealth. The charity is therefore well-placed to lead essential discussions on medicines access, medicines supply and pharmaceutical care, as well as the broader ways pharmacists can support access to health and health information in their communities.

This commentary discusses the key medicine-related issues that are facing the Commonwealth today, and summarizes how the CPA can support member nations to achieve UHC and more equitably and effectively serve the health needs of their population in the midst of a pandemic, in line with the five CHOGM subthemes and the global health agenda.

## Information and communications technology (ICT) and innovation

With the current restrictions on face-to-face social interactions and travel, there is an urgent need to explore the opportunities that ICT can provide in enabling more innovative and flexible models of pharmaceutical care delivery. However, we should be aware that ICT should help address and not add to existing inequities both within societies and between LMICs [13]. The use of electronic or faxed prescriptions and ‘mail-order’ or ‘home-delivery’ pharmacy models to facilitate remote healthcare are all potential solutions that can help ensure ongoing pharmaceutical supply to patients whilst minimising the need for in-person interactions [8, 14]. However, the technology required to support these models of care delivery are not fully integrated in some countries, including many LMICs. As pharmacies in other higher income Commonwealth countries such as the UK, Australia and NZ move towards these new models of delivery [15], we must ensure that LMICs are not left behind. There is reassuring evidence to suggest this will not be the case. With increasingly widespread mobile phone use, African nations have pioneered in mobile banking, which is widely used across the continent since mPesa was established in 2007 [16]. Initiatives, such as the launch of the Ghanaian COVID-19 tracker app [17] follow this trend of digital innovation.

ICT offers many opportunities to increase access to healthcare and support everyday practice by pharmacists. Low-cost innovations such as Mobidawa and WelTel mobile phone technologies to disseminate health messages, for example specific information on how to take medication, supports communication with traditional ‘hard-to-reach’ communities in Kenya and Cameroon [18]; increasingly African network providers are sending public health information and alerts such as reminders to use malaria nets during national prevention week.

ICT also exists to aid the detection of substandard and falsified (SF) medicines [19]. There is a large evidence base demonstrating widespread problems with poor quality antimicrobial drugs and other essential medicines in Southeast Asia and sub-Saharan Africa [20, 21]. The COVID-19 pandemic only serves to exacerbate such issues, including the risk of misinformation and false claims, for example through the use of SF medication for preventing and treating COVID-19 [22, 23]. Poor quality medicines are a significant barrier to UHC and undermine efforts to improve healthcare [24]. Consequences of using SF medicines include antimicrobial resistance, adverse drug reactions, toxicity, and treatment failure [19]. With the COVID-19 pandemic, there is an increased urgent need to fast-track the development of technologies to counter falsified claims, detect poor quality medicines and save lives, as well as share innovation in this area. For example, the MPedigree barcode scanning app has been implemented in several African countries to allow checking of the validity of medicines [25]. Additionally, new technologies such as PharmaCheck, which uses a handheld scanner to analyse medicines, offer competitive new ICT solutions [19] to build regulatory technical capacity. Medicines quality assurance requires a robust regulatory system overseen by a medicines regulatory agency, however in many LMICs this is limited by resource and capacity constraints. However, pharmacoviligance reporting apps developed by regulatory authorities in Ghana [26] and Zambia [27], highlight how digital innovation can enhance regulatory systems. Real-time ‘point of sale’ reporting devices or mobile apps such as ‘MedSnap’, a mobile phone app that is capable of identifying medications, and assist with the data collection and monitoring of SF medicines [28], is currently being trialled by the CPA in partnership with the Pharmaceutical Society of Zambia. This is intended to synergise with the pharmacoviligance reporting app developed by the FDA to build greater medicines regulatory capacity.

In tandem to regulatory strengthening, it is imperative to ensure patients have access to a quality assured supply route [29]. In Rwanda, innovative approaches using drones have been applied to increase medicines access in remote regions [30], which are now being extended to West-African regions. In Ghana, pharmacists are innovating apps to increase medicines efficiencies and patient access to vital medicines [31].

ICT also has a pivotal role to play in countering the COVID-19 ‘infodemic’ [32]. Shared common information platforms, such as the online resource portals created by national pharmacy associations and international organisations such as the CPA and International Pharmaceutical Federation (FIP), can facilitate the timely exchange of news, alerts on outbreaks, opportunities for collaborations and support continuing professional development (CPD) between pharmacists. To facilitate this, regional and national policies and ICT linkages need to be established, as well as infrastructure to support access to stable internet and connectivity particularly in rural and deprived areas. CPD to improve pharmacist technology literacy will also need to be prioritised [33]. For example, in Zambia, there is an increasing demand for pharmacy services, with growing numbers of pharmacists keen to upskill and evolve new roles to expand the breadth of the profession. In line with a recent change to mandatory CPD, the CPA have worked closely with Zambia to explore the opportunities for delivering CPD via an online learning platform to address pharmacist training gaps. Many medicine information resources and centres are online, which can act as repositories of evidence-based information. However, in many healthcare settings, internet access can be intermittent and there is a need for easy access to key information, compiled in one place that can be made available offline. A successful example of this is the Commonwealth Partnerships for Antimicrobial Stewardship (CwPAMS) app which supports the rational use of antimicrobials, beginning with Ghana, Tanzania, Uganda, and Zambia [34].

Innovations in ICT can facilitate the better use of medicines by enabling pharmacists to provide data-informed practice, such as software to manage medicines dispensing and inventory [35] and technologies to check culture sensitivities, identify pathogens and aid diagnoses prior to the supply of antibiotics to support antimicrobial stewardship initiatives [36, 37]. Ensuring the right antimicrobials are used for secondary infections where indicated, but not inappropriately used for COVID-19, is particularly relevant; the current CwPAMS prescribing app is an example of an innovation that has developed COVID-19 specific resources to support this [34]. Indeed, the time could not be more apt to be embracing digitalisation. At the 2019 annual conference of the Pharmaceutical Society of Ghana, the Vice President of Ghana called for pharmacists to embrace digitalisation, enhancing patient recordkeeping and medicines access, noting their expanding and evolving role in supporting a health system moving towards UHC [31].

## Trade

Exploring the impact of the current COVID-19 pandemic on issues of medicines shortages and ensuring ongoing access and supply of medicines is a key priority. Even prior to this global pandemic, there have been calls for a coordinated approach to address issues of medicines access and the need for reporting systems for medicines shortages. This was raised in the Report of the International Summit on Medicines Shortages in 2013 hosted by the FIP and the Canadian Pharmacists Association [38], with models and tactical options to address shortages summarised in a 2017 report by FIP [39].

With medicines being one of the most important interventions in healthcare, ensuring continuity of medicines supply with the current trade and travel restrictions in place as part of the pandemic response is key. There needs to be timely and accessible ways of monitoring and reporting on medicines access issues, with a global process to produce a list of critical or vulnerable medicines for each country. We do not yet fully know what the impact of the COVID-19 pandemic will be on trade and supply of medicines, including raw materials – there needs to be urgent research in this area to inform how we can support Commonwealth countries to deliver essential healthcare. This knowledge of the vulnerabilities in our medicines supply process will remain useful beyond the pandemic, as issues of access and affordability are relevant to any major global and political changes, such as with Brexit [40].

There is great potential for local production of medicines to allow tighter regulatory control and support better quality assurance and supply of essential medicines, as a result of a more efficient use of resources, and efficiency of time and supply, particularly of temperature sensitive medicines such as vaccines. Local manufacturing can support the provision of more cost-effective quality treatments, such as has been recognised by several member countries. The Commonwealth can achieve this by promoting local manufacturing capacity through supporting local companies to become Good Manufacturing Practices (GMP) compliant, establishing bioequivalence centres to support the local industry, providing financial support (e.g. long-term, low-interest patient capital), and linking to other countries with strong local manufacturing capacity (e.g. India, Malaysia, Bangladesh, Kenya, Ghana), which can help increase global competitiveness. For example, in Ghana, initiatives to foster a manufacturing ‘hub’ are anticipated to foster economic growth in the pharmaceutical sector and improve access to quality assured medicines. This includes tax exemption on raw materials and essential medicines produced locally and a 15% local price preferment strategy [41]. In tandem, the Ghana pharmaceutical sector development strategy includes capital loans to incentivise producing medicines on the national health insurance list [42].

Current barriers to exporting locally manufactured generics relate to the risk of SF medicines; training to identify these low-quality medicines such use of serialisation to trace and track and regulation to streamline trade, needs to be prioritised. Calls for regional harmonisation and initiatives to pool regulatory efforts, such as through the African Medicines Regulatory Agency [43], have highlighted significant progress [44]. Yet, further work is also needed to implement the Continental Free Trade Agreement to iron out modalities of free trade in pharmaceuticals amongst African countries. This is particularly pertinent in areas such as cancer, where intellectual property and the expertise required to develop complex medicines remains with few manufacturers [45].

Linking in with ICT, the sharing of information on product innovation can facilitate discussions on how to tackle the challenges of different trade policies, currency, language/cultural barriers; and allow a common platform for free information exchange. Use of a pooled procurement strategy to facilitate shared access to pharmaceuticals can also increase medicines accessibility by improving the efficiency and affordability of their procurement [46].

## Environment

With an increasing global population, the notion of ‘Green pharmacy practice’ is an essential topic for discussion. Considering how pharmaceuticals impact on the environment at both the manufacturer and individual level is important [47] – for example, ensuring responsible use of medicines to reduce wastage, managing the safe disposal of unused / unwanted / expired medicines, and the use of plastic-free packaging options. This helps to safeguard sustainable practice and overall responsibility to uptake methods to reduce the environmental impact of medicines [48]. Disposal of medicines is also costly with some countries facing fees charged by regulators to dispose medicines, or high costs to use incinerators to safely dispose pharmaceuticals [49]. Countries need to move towards free disposals to encourage more responsible disposal, for example by developing guidelines and allocating funding to provide the needed infrastructure to support safe disposal of medicines, green and eco-friendly manufacturing, and campaigns to promote public awareness [50]. Of particular importance is the need to consider safe disposal of antimicrobials, as inappropriate disposal, for example of antibiotics into waste water [51], can worsen antimicrobial resistance.

## Good governance and the rule of law

The significance of pharmacies and pharmacists has been brought to the forefront in the recent pandemic, with many pharmacy services around the world overwhelmed [52]. Never has the world seen such a focus on pharmacy service provision; the advantages of the dual-role of community pharmacists as retailer and health provider have emerged [53] as countries have gone into lockdown and pharmacists are named as a ‘vital’ part of the healthcare system as one of the few essential services that can remain open to the public. Beyond the pandemic, we need to ensure that this significance of pharmacists as an essential health provider is not diminished. There needs to be ongoing support at the national and global level for pharmacy services to be implemented in their full potential. This is particularly important when considering the shortfall of 18 million health workers by 2030 that is projected by WHO and the amplification of this problem in LMICs [6]. Pharmacists are skilled worldwide to provide more extensive public-facing services – such as family planning, HIV testing, screening, and vaccination. These extended pharmacy services – particularly vaccination services – will be key for achieving population vaccination coverage when a COVID-19 vaccine becomes available, especially as pharmacies are one of the most accessible health providers in the community [54]. The effectiveness of utilising pharmacists for public health interventions has been highlighted through Commonwealth initiatives led by female pharmacist groups to set-up clinical breast screening and raise awareness to reach the most vulnerable communities. This would help with healthcare access for the public yet policy-level barriers need to be overcome to enable full utilisation of the pharmacy workforce globally, as well as cold-chain issues.

New policies may be needed to support the evolution of pharmacy practice, for example the establishment of senior pharmacist roles at the government level, and involvement of national pharmacy associations in the creation and implementation of health policies. Pharmacists should be key stakeholders on all discussions involving medicines if systems and policies are to be well thought out and effective. Governments need pharmacist skillsets, but steps need to be taken to strengthen the profession and ensure they are appropriately regulated and recognised. This good governance facilitates better regulation of medicines, pharmacists and pharmacies. Without legislation or governance that specifically defines pharmacist roles and pharmacy practice, it is difficult to advocate for the expanding role of pharmacists. For example, in Zambia, the pharmacy profession is not currently self-regulated despite 60% of budget being spent on pharmaceuticals. There is a need for pharmacists to have their own pharmacy council to clarify roles and functions of regulatory agencies, whilst preventing overlap in functions leading to over-regulation and associated financial burdens. Pharmacy councils are needed to address issues at a national level collectively via implementation of regulations. For example, the Pharmacy Council and Pharmaceutical Society of Ghana, together with Management Sciences for Health (MSH), conducted a pharmaceutical service mapping exercise of a rural region in Ghana to highlight gaps to improve access to quality assured medicines. Other examples of issues where collaborative actions are required include SF medicines and its contribution to AMR, ensuring honest and corruption-free systems, and reviewing licencing systems so licenses to practice are based on a minimum required level of skill and knowledge to ensure public safety. For example, community unregulated sale of medicines, such as at markets and stores, in particular antibiotics, are of high concern in many LMICs [55]. The CwPAMS has brought together practitioners and policy makers and raised these issues leading to acknowledgment and action by pharmacy councils to work with unlicensed sellers of antibiotics to address these issues [34]. There is potential for the CPA to support this by creating online knowledge sharing symposia, webinars or online workshops.

## Youth

Encouraging youth to be the next generation of health professionals needs to be high on the Commonwealth agenda as the numbers seeking to study medicine, nursing and pharmacy begin to decline whilst the public health needs increase. In the UK, the undergraduate university courses for medicine, pharmacy and nursing have been undersubscribed; there is a need to understand the drivers of this to increase enrolment and ensure ongoing workforce development, equity and diversity. Engaging with the youth in policy development and stakeholder representation – for example through establishing local and national Young Pharmacists Groups and linking with the Commonwealth Youth Network, WHO Global Health Workforce Network (Youth Hub) – are key first steps. The different mindset and priorities of the youth generation needs to be considered – innovative ways to entice young people in key health professional roles are needed. Many youth enjoy portfolio working and travel opportunities – knowledge and skill exchanges such as twinning and health partnership programmes between LMICs and other countries, for example the CwPAMS scheme [34], may be particularly attractive to youth. Public education, campaigns and mentorship programmes to support youth development as future health leaders are needed.

Simultaneously, many countries across the Commonwealth have young populations and the pharmacy workforce is dynamically evolving and rapidly expanding [11]. At the national and global level, governments and health organisations need to ensure that any increase in number in the pharmacy workforce is matched by an increase in pharmacy roles and job vacancies. For example, in Zambia, there are nearly 200–300 new pharmacists each year, in Rwanda the number of pharmacists has grown rapidly after the establishment of the Rwanda Community Pharmacists’ Union in 2016 [56], and in Tanzania, there are over 1900 pharmacists, including 500 hospital pharmacists; with such rapid growth in the pharmacy profession, there is a need to ensure jobs and roles for new graduates. This will include in clinical settings in hospitals, where pharmacists have key roles in ensuring rational medicines use as part of multidisciplinary team. In many LMICs, pharmacists still hold a traditional supply role and clinical pharmacy is at a nascent stage [5]. Skill sharing initiatives such as CwPAMS with settings where clinical pharmacy has been well-established have led to expanding pharmacists’ roles and cultivating a culture of clinical pharmacy, gain policy-leader interest. The CPA are currently exploring approaches to expand and sustain this through sharing education and skills to meet the demands of the growing workforce and population health priorities. However, in certain countries, there is a current lack of employment in the public sector, and the lack of funds available to pharmacists to support the establishment of businesses are perceived as barriers. It is critical the momentum and energy behind the growing pharmacy profession is capitalised on to support progress towards SDG 3 and beyond.

### Summary

The current global crisis that the world is facing lends itself to new challenges and opportunities for the pharmacy profession. The time is now for the Commonwealth to capitalise on the diversity within its network to work together to respond to these challenges and consider how the pharmaceutical workforce can be positioned to support the global COVID-19 pandemic response, and beyond that post-pandemic, to progress towards UHC. The CHOGM 2020 themes provide a timely and useful framework to consider our global response. The pandemic has highlighted the crucial role of pharmacists in essential healthcare delivery; the significance of the pharmacy workforce in healthcare needs to be continually recognised beyond the pandemic as global health needs increase and change. Together we must unite in the fight against this and future global health crises and ensure equal health outcomes for all.
